# Primary healthcare training in the Democratic Republic of the Congo

**DOI:** 10.4102/phcfm.v16i1.4508

**Published:** 2024-05-24

**Authors:** Jean-Pierre Fina-Lubaki, Junior Mudji E’kitiak, Philippe Lukanu Ngwala

**Affiliations:** 1 Department of Family Medicine and Primary Care, Faculty of Medicine, Protestant University of Congo, Kinshasa, Democratic Republic of the Congo

**Keywords:** primary care, family medicine, training, education, challenges

## Abstract

Family medicine is a relatively new discipline in the Democratic of the Congo. It was developed under South–South and Churches Collaboration with the aim of responding in a cost-efficient manner to the crisis of health practitioners in mostly Christian and protestant hospitals based in rural areas in the Democratic Republic of the Congo.

## Introduction

**FIGURE 1:**
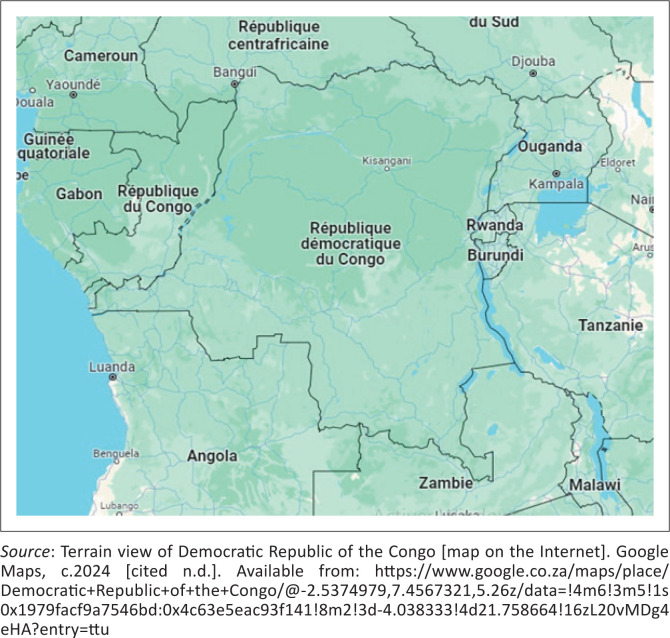
Democratic Republic of the Congo.

The Democratic Republic of Congo (DRC) is located in Central Africa and covers an area of 2 345 409 square kilometers (Figure 1). The country has an estimated population of 99 million, with an annual population growth of 3.2%.^1^

The country is divided into 26 provinces and is managed on a highly decentralised unitary basis. The population is very young, the majority are poor and without social protection.^2^

The DRC is classified as a low-income country and spends only 4.3% of the gross domestic product on health.^1^ Household contributions to healthcare costs from out-of-pocket payments are still very high, up to 43%.^3,4,5^

The Congolese healthcare system takes the form of a pyramid with three levels. The central level at the Ministry of Health is responsible for issuing standards.^6^ The intermediate level supervises activities within provinces. At the primary level, the health zone or district is the operational unit – the country has a total of 516 health zones. Each health district is managed by a district management team consisting of the head of the health district – a generalist most of the times – assisted by administrative staff and technical supervisors.^7^ The district management team supervises the activities of the healthcare facilities. Usually each health district has one hospital surrounded by about 20 health centres.^7^ The health centres are managed by generalists or even nurses in rural areas. Mainly in Kinshasa or a number of towns in the country specialists could be found at the secondary or tertiary levels of care. There is an estimated 1.2 doctors, nurses and midwives per 1000 population.^8^

The DRC is currently facing the double burden of communicable and non-communicable diseases.^7^ Several diseases with epidemic potential (cholera, measles, yellow fever, Ebola virus disease) are emerging and re-emerging. Endemic diseases include malaria, HIV/AIDS and tuberculosis. The health system is mainly organised for child and maternal care and tackling communicable diseases. The health centre offers a minimal package of services that include primary curative care, promotional and preventive activities. The hospital offers, apart from the minimal package, a complementary package of services, which consist mainly of basic surgical, obstetrical, gynaecologic, internal medicine and paediatric care.

The three priority issues in the health system are problems related to the provision of health services (low coverage, poor quality, low utilisation of health services, no preparedness for emergencies and disasters or non-communicable diseases), health system support (inequitable distribution of healthcare professionals, poor infrastructure and equipment, low availability of medicines and disposables, low allocation of public resources) and the governance and management in the healthcare sector.^7^

As a consequence, the country’s key health indicators are poor (maternal mortality rate: 547 [377–907]; infant mortality ratio: 62.37 [39.76–93]).^1^ The latent war in the eastern part of the country prevents improving health for the populations concerned.

## History of family medicine training in the Democratic Republic of the Congo

Primary healthcare (PHC) is the most inclusive, equitable, cost-effective and efficient approach to enhancing people’s physical and mental health, as well as social well-being.^9^ Moreover, PHC is the cornerstone of universal health coverage.^10^ One priority in reinforcing primary care is to train family practitioners.

The history of family medicine in the DRC started with the Church of Christ in Congo, Zaire in 1985, which was involved in co-managing the health of the population in the health zones where it was established. As most of our facilities are located in rural areas, where the population has little access to specialists, the Church of Christ in Congo wanted its health facilities to be staffed by general practitioners with enhanced skills, capable of responding to the population’s day-to-day needs. Young physicians were trained in a holistic approach to the practice of medicine.

In 1997, the Church of Christ in the Congo signed a partnership with the Medical University of South Africa, currently Sefako Makgatho Health Sciences University (SMU), to train Congolese doctors in family medicine. As a result of this partnership, and with the help of Evangelischer Entwicklungsdienst (EED), a German church protestant-based organisation, the Protestant University of Congo developed a family medicine training programme between 2011 and 2014. This is still the only university to offer postgraduate training in family medicine.

In recognition of the quality of care offered and taking into account the benefits that could ensue from family medicine in the Congolese PHC system, the Ministry of Higher Education recognised the training in 2011. In 2021, family medicine was registered as a specialty by the Congolese Medical Council (CMC).

## Training in primary care and family medicine

The DRC has about 57 faculties of medicine. At all these universities, at the undergraduate level, first year medical students are offered 20 h of theory and 1-month holiday placement in primary care. Only at the Protestant University of the Congo is a 15 h module offered to fourth-year medical students on the principles of family medicine, community-based healthcare and ethics.

At the postgraduate level, since 2011, with the support of the SMU and other partners, The Protestant University of Congo has organised a 4-year training course in family medicine, and currently, about 60 family practitioners have successfully graduated. The training consists of 3 years of clinical rotation in the main clinical domains (paediatrics, internal medicine, surgery, obstetrics and gynaecology, emergency medicine, ophthalmology, dental care and PHC) and 1 year of research.

The role of family physicians has still to be defined in the health system. The trained family physicians are employed mainly as generalists in the private or public sector; a number work for non-government organisations or have emigrated. Their payment varied widely.

## Challenges and future perspectives

Family medicine is a relatively new medical discipline in the DRC, and the fact that it is not recognised as a specialty in many Francophone countries has been a real barrier to the uptake of the discipline in the country.

Nevertheless, after 13 years of teaching family medicine at the Protestant University of the Congo, and with more global recognition of family medicine, the idea has been spreading in the medical community, and one can see an increasing interest among young doctors in pursuing the discipline. There is a clear need to advocate for an extension of the training in family medicine to other universities.
